# Communication Processes and Priorities in Medical Aid in Dying Conversations: A Cross‐Sectional Qualitative Study of Multidisciplinary Cancer Clinicians

**DOI:** 10.1002/cam4.71762

**Published:** 2026-04-02

**Authors:** Meghan McDarby, Alix Youngblood, Megan Miller, William E. Rosa, Haley Buller, Betty R. Ferrell

**Affiliations:** ^1^ Department of Psychiatry and Behavioral Sciences Memorial Sloan Kettering Cancer Center New York New York USA; ^2^ Department of Medicine Memorial Sloan Kettering Cancer Center New York New York USA; ^3^ School of Nursing University of Wisconsin Madison Madison Wisconsin USA; ^4^ City of Hope California USA

**Keywords:** assisted dying practices, cancer communication, end of life, end‐of‐life communication, hospice, medical aid in dying, multidisciplinary, palliative care, person‐centered care, physician‐assisted suicide

## Abstract

**Background:**

Medical aid in dying (MAiD) is a practice that enables eligible individuals with a terminal, life‐limiting illness to end their lives in a self‐directed way. Multidisciplinary care teams play a vital role in facilitating discussions and patient decision making about MAiD in cancer care settings. However, little is known about how multidisciplinary care teams navigate MAiD discussions to provide effective, person‐centered care.

**Objectives:**

To identify the communication priorities and processes that cancer care clinicians believe are most critical for multidisciplinary care teams when discussing MAiD with patients who have cancer.

**Design:**

Cross‐sectional online survey.

**Setting/Subjects:**

Multidisciplinary clinicians who participated in a communication training program supported by the National Cancer Institute (*N* = 160) responded to five open‐ended survey questions about communication considerations when working with a hypothetical patient with glioblastoma considering MAiD. Participants were presented with a case scenario and invited to describe how they would communicate with that patient as a member of a multidisciplinary team. A thematic analysis of responses using an iterative, inductive approach was conducted until saturation was reached.

**Results:**

Four themes were identified as communication priorities and processes critical for multidisciplinary teams when discussing MAiD with cancer patients: (1) addressing complexity of MAiD in GBM; (2) thorough palliative care assessment; (3) strategies for clinicians and healthcare systems to optimize MAiD discussions; and (4) person‐centered care that de‐stigmatizes MAiD.

**Conclusions:**

Findings underscore the distinct complexity of MAiD discussions in oncology and highlight the need for tailored, person‐centered approaches that go beyond standard end‐of‐life communication.

## 1 Background

Medical aid in dying (MAiD) is a practice in which eligible individuals with a terminal, life‐limiting illness can request and receive medication to end their lives in a self‐directed way [1]. MAiD has been the subject of significant scientific and policy debate related to clinician ethics and duty, patient trust and autonomy, risk for vulnerable populations, potential for coercion and abuse, and the argument that increased access to hospice and palliative care services should be prioritized. Globally, clinicians disagree on the fundamentals and implications of MAiD. One survey of 263 international multidisciplinary clinicians in 66 countries showed that about half support the availability of physician‐assisted suicide (i.e., MAiD) [2], and 56% of those in favor believe that it should be used only in specific and exceptional situations [3, 4, 5]. In 2025, MAiD was legal in 12 states in the United States, and MAiD legislation was under consideration in another 17 states [6], affording roughly 22% of the United States population access to MAiD [7]. As a result, MAiD has become increasingly relevant in oncology settings, as more states nationally and other countries adopt legal frameworks permitting its use and outlining recommendations and guidance for ethical applications at end of life (EOL) [8].

Although MAiD is available to patients with a range of life‐limiting illnesses, including neurological diseases, the majority (74%) of MAiD recipients are diagnosed with advanced cancer [7]. Patients with cancer often seek MAiD at EOL due to high symptom burden [9] and distress [10], with some estimates suggesting that as many as 70% of oncologists have cared for a patient seeking MAiD [10]. For patients with advanced cancer, conversations about MAiD are logistically and ethically complex, situated within the broader context of EOL decision making [11, 12]. Given the impact of cancer and cancer treatment on quality of life for many patients [13, 14], discussions about MAiD must take into consideration a patient's motivation to participate in MAiD, including an in‐depth evaluation of their emotional, mental, social, and financial wellbeing, and incorporate a complete assessment of prior measures taken by both the patient and the cancer care system to alleviate suffering and improve patient quality of life [15, 16]. Furthermore, MAiD discussions with cancer patients require careful navigation to ensure that patients' values, goals, and needs are honored [17], and that the perspectives of family members and other care partners are fully respected and appropriately integrated into care discussions [18, 19].

Multidisciplinary care teams (MDTs) play a vital role in facilitating MAiD‐related conversations in cancer care settings [20]. MDTs in oncology typically include physicians, nurses, social workers, chaplains, psychologists, and can also include other allied health professionals (e.g., dieticians, physical therapists) [21]. Each discipline brings a unique perspective to patient care in oncology, and when operating optimally, contributes in a way that complements the knowledge, expertise, and skillsets of other members of the MDT [22]. However, while diversity of professional disciplines has the potential to enhance the quality and breadth of support available to patients with cancer nearing EOL and contemplating MAiD [23], it also introduces complexity into communication processes [24]. Misalignment or unclear roles can lead to conflicting messages, ethical tension, or unintentional harm [25]. Furthermore, lack of clarity about the individual responsibilities of each provider on an MDT in MAiD discussions and the responsibilities of those clinicians to honor MAiD legislation as it pertains to different jurisdictions can have significant negative consequences on upholding best practices of person‐centered care [26, 27]. Understanding how teams navigate these challenges is essential to developing best clinical practices that are both multidisciplinary and person‐centered. However, little is known about how care teams can consistently uphold these principles across varying clinical roles and contexts during MAiD conversations.

The purpose of the current qualitative study was to identify the communication priorities and processes that are most critical for MDTs when discussing MAiD with patients who have cancer. We intentionally chose to situate the project around a case study of a patient with glioblastoma (GBM), as treatment for GBM is inherently multidiscliplinary [28], has a relatively predictable poor prognosis [29], and has diagnostic complexities (e.g., cognitive decline, changes in communication) [30] to increase opportunities for participants to consider the intersection of a complex cancer diagnosis with challenging communication. By examining how different team members contribute to and experience these conversations, we seek to inform the development of more cohesive, compassionate, and coordinated communication strategies. Ultimately, this research will contribute to the growing body of evidence guiding ethically sound and patient‐centered care in MAiD contexts, particularly within oncology.

## 2 Methods

### 2.1 Participants and Procedure

Participants were 160 multidisciplinary oncology clinicians from the United States who care for adult oncology patients (Table 1). Approximately one third (36%) of study participants lived and worked in one of the 12 states where MAiD was currently legal at the time of their response. Prior to participation in the study described herein, participants attended a 3 day, in‐person communication skills training program funded by the National Cancer Institute (NCI) titled the Interprofessional Communication Curriculum (ICC) Project [31, 32]. The ICC is organized around the National Consensus Project for Quality Palliative Care clinical practice guidelines [33]. After completing the course, participants from two unique cohorts of the ICC were invited to complete a short survey. We distributed the survey to two unique cohorts of the ICC course to capture responses from a large, broad, heterogeneous, and nationally‐representative sample of oncology clinicians. Participants provided consent to participate in the survey as part of the initial ICC Project application process. Participants from the first cohort completed the survey immediately following the 3‐day course (51.9%; *n* = 83); participants from the second cohort were invited to complete the survey 12 months post course (48.1%; *n* = 77). Study procedures were deemed exempt by the City of Hope IRB, since all data were collected from course participants only and could not be linked to individual respondents (assurance number 00000692).

**TABLE 1:** Participant demographics (*N* = 160).

	Total	%
Sex		
Female	144	90
Male	16	10
Ethnicity		
Hispanic	28	17.5
Non‐Hispanic	132	82.5
Race		
American Indian/Alaskan Native	1	0.6
Asian	8	5.0
Black or African American	17	10.6
More than one race	13	8.1
Native Hawaiian or Pacific Islander	2	1.3
White	119	74.4
Professional Discipline		
Nurse	74	46.2
Social Worker	54	33.8
Chaplain	24	15.0
Other (physician, doula, psychologist)	8	5.0
States		
MAiD is Legal	58	36.2
MAiD is not Legal	102	63.8
Work Setting		
Ambulatory/Outpatient Cancer Care Center	24	15.0%
Community Cancer Center (Clinical or Comprehensive)	20	12.5%
Hospital/Oncology Unit	63	39.3%
NCI Designated Cancer Center	31	19.4%
Other (e.g., hospices)	22	13.8%

### 2.2 Measures

Participants reported demographic information, including race, ethnicity, state of practice, and professional discipline. Then, participants responded to five open‐ended questions about a case of a 57 year‐old individual recently diagnosed with GBM who is seeking MAiD due to concerns about loss of autonomy, dignity, and quality of life (Table 2). We specifically selected a case about GBM as we knew it would raise important ethical, emotional, and clinical considerations for clinicians around EOL communication in cancer care settings. For example, patients with GBM who are interested in learning more about MAiD, like the patient described in the case study, are likely to raise questions about MAiD early or even at the point of initial diagnosis, given its relatively predictable prognosis and course. Further, compared to communication in other cancer types, EOL communication in the context of GBM is additionally complex given changes that patients experience in verbal communication abilities as the disease progresses. Lastly, given the nature of the progression of GBM and the complexity of its symptoms for patients, it often evokes more complex emotions for clinicians, creating more significant challenges in communication.

**TABLE 2:** Case Study and Corresponding Questions.

Reese is 57 years old and was diagnosed with glioblastoma last week. Since their initial diagnosis, Reese has expressed a strong desire to obtain a prescription that would help them end their life before the symptoms associated with their glioblastoma became too overwhelming. Reese is afraid of losing control of their body (e.g., incontinence) and is also afraid of “not feeling like themselves” (e.g., mood swings, memory loss). They also have a strong feeling that they do not want to keep living if they are not able to do the things they enjoy. Reese has requested to be a recipient of Medical Aid in Dying, or MAiD. Reese has already mentioned this preference to their primary oncologist who has not pursued the matter further—they want Reese to take at least 2 more weeks to think about this decision before moving forward with the MAiD evaluation process. Imagine that you are caring for Reese today and they raise this topic of MAiD, again voicing a strong desire to pursue MAiD and their desire for life to end before symptoms become too burdensome	What questions would you ask Reese to better understand their desire to pursue MAiD? How can you ensure that you communicate with Reese in a non‐biased, non‐judgmental way, regardless of your own personal opinions about MAiD? Previous discussion has often focused only on the role of physicians in communicating with patients about MAiD. What are the roles of all members of the interdisciplinary team in responding to a patient who wants to pursue MAiD? If you have communicated with a patient about MAiD, can you share your experience in having these conversations? What was most challenging? What was most helpful or meaningful? Are there other issues that you think are important regarding communication about patient requests for MAiD? How can clinicians work as interdisciplinary teams to support each other and patients/families when communicating about MAiD?

### 2.3 Data Analysis

A thematic analysis was conducted using an iterative, inductive approach. Coding was completed by two research team members (MM, AY). WER supported early discussions about coding and participated in discussions about the approach to achieving consensus. During early coding, authors (MM, AY) met and used a consensus approach to apply codes to individual participant responses. The coding team repeated this process for all participant responses to each question until an understanding of how to apply codes had been reached. MM and AY then equally divided and independently coded the remainder of responses. Coders met regularly throughout the coding process to share findings and resolve questions about coding. Codes were organized into themes, and key properties of each theme were identified across questions. Thematic saturation was reached and was established based on criteria of forcefulness, repetition, and recurrence [34]. We did not code participant responses to individual survey questions when they were not substantive (e.g., “I don't know,” or “None”) or when they pointed to a participant not being able to answer the question of interest (e.g., “I have never worked with a person requesting MAiD.”). Coding was conducted using Dedoose Version 9.2.22.

## 3 Results

We identified four themes characterizing participants' (*n* = 160) perceptions of communication priorities and processes that are most critical for MDTs when discussing MAiD with patients who have cancer: (1) addressing complexity of MAiD in GBM; (2) thorough palliative care assessment; (3) strategies for clinicians and healthcare systems to optimize MAiD discussions; and (4) person‐centered care that de‐stigmatizes MAiD. Table 3 describes themes, as well as specific properties of each theme and illustrative quotes.

**TABLE 3:** Meaningful Experiences: Themes, Thematic Properties, and Illustrative Quotes.

Theme	Thematic property	Illustrative quotes
1. Addressing complexity of MAiD in GBM	Support patient conceptual understanding of MAiD	I think it is important for all patients to be well informed about the law and have access to resources that can help them access the aid in dying medication (P138, professional discipline unknown). Reese, can you tell me more about what the doctors told you? What do you already know about MAID in this week since your diagnosis? (P124, nurse). My role primarily has been to educate regarding MAID. After education if patient continues to express desire to pursue their rights under MAID, I refer to our specialized team to initiate process (P92, social worker)
	Assessment of fears and fear of the future	I would ask Reese about their fears regarding their illness, death, and dying (P52, chaplain). Can you tell me what your fears are? Can you tell me what you would like your death to look like? (P34, nurse). What does living mean to you? What does it mean to you to die with dignity? (P119, nurse)
	Understand drivers of MAiD interest/preference	So much information about your body has been shared with you recently. It is understandable to want to take ownership of what you can. You shared some fears of potential physical changes in the future, have you ever had a time in your life where you felt you couldn't control what was happening to you or your body? What did you or your supports to help at that time? (P134, social worker). Reese, tell me more about your fear of losing control of your body (and or the fear of not feeling like themselves). What scares you the most? (P132, chaplain). If symptom control for the resulting health issues related to the glioblastoma could be addressed medically, would that influence her decision about MAID? (P16, social worker). Is there a reason why he would not want to wait 2 weeks before deciding? (P140, nurse)
2. Thorough palliative care assessment	Psychosocial assessments to understand role of support network	I would ask Reese what their support at home looks like, I would dive deeper into understanding Reese's feelings of needing more assistance, I would find out what things Reese likes to do and ask if there are reasonable accommodations they think family or friends could make to help them live with quality. I would ask Reese if they needed assistance talking with family/friends about their desires, and also ask about them having their affairs in order (P154, nurse). What things bring you joy? What is most important to you? Do you have any history of mental health issues? What is your support network life? What symptoms worry you the most? (P96, social worker). Trying to learn/understand about the patient's experience and emotions as well as beliefs and important connections and offering emotional validation was very important. I listened a lot before I spoke. They felt heard, and by the end of the discussion shared they felt more hopeful to keep living (P40, chaplain)
	Spiritual care and values assessment	Tell me more about your symptoms and how they affect you, not just physically, but mentally, emotionally, and spiritually? Tell me a bit more about yourself. Are you part of a faith community? (FICA/CASH assessments) (P150, chaplain). Does the patient belong to a specific faith‐based religion and what are the rules according to their belief system? (P26, chaplain). Do you consider yourself a spiritual person? (P40, chaplain)
	Explore uncontrolled symptoms	In the case with intractable pain however, this led the patient and family to take on discharge from the VA to explore, proceed and complete the process on their own as a option for sx management vs. fear (P134, social worker). What symptoms are you concerned with and what would you feel is too burdensome? (P128, nurse). I would ask more detailed symptom management questions and suggest we form a collaborative team to specifically discuss concerns incl. pt.'s Med Onc., Pall Onc, family and RN and to form a plan together that provides for current needs, as well as address pt.'s future wishes (P116, nurse)
	Assess prognostic understanding	Do they understand all options for end of life, i.e., palliative care, hospice, comfort care? Do they understand her prognosis and treatment options? i.e., does she have 6 months or less? (P146, social worker). Can you tell me more about your understanding of where you are and your illness? (P47, social worker). It is important that the patient has an accurate understanding about their prognosis, possible symptoms, and potential for living with support necessary. Clinicians ideally would come to an agreement on standards of practice within an organization regarding what is communicated to patients and families (P42, nurse)
3. Strategies for clinicians and healthcare systems to optimize MAiD discussions	Increase self‐awareness as a provider	Check in on my own emotional and reactionary response to any of the circumstances of the case. Ask open ended questions, that are not leading, but reveal Reese's meaning and values (P84, chaplain) (1). I ground myself before I go into the conversation (2). I open my mind to whatever he needs to express knowing that I don't have to make any decisions (3). I settle into gathering information in a way that I can clearly relay it to whomever will support him now or later and make sure I chart everything we talk about (P75, professional discipline unknown). I would take a few deep breaths to center myself and release my own emotions/beliefs before continuing the conversation (P51, chaplain). I set my own initial thoughts about MAID aside and begin to understand the person and their values and spirituality (P157, chaplain). I will make sure that I am not bringing my own personal believes into the conversation. That this isn't my decision whether they pursue MAID and that there is also a process that they will have to qualify for MAID and that ultimately that is out of my hands (P151, social worker). Every member of the team have [sic] to be comfortable with allowing patients to make their own choices. This starts with examining our own beliefs and values (P86, social worker)
	Improve and promote education about MAiD policies and procedures	What might be challenging for me is if the patient opted not to seek treatment at all and instead chose to do MAID. What is most helpful is attending and participating in courses such as these. The more exposure one has to this the more comfortable it'll be during these times (P154, nurse). Providing education to nursing staff/primary doctors is important too there is a lot of misconceptions about the process and the law (P151, social worker). I believe that all staff need to be familiar with MAID. I think staff will say to not think about that in an effort to reassure the patient they will be okay and don't have a comfort level of discussing MAID. I would like to see specific training for the different disciplines to address this topic with patients if they bring it up and realize that patients can have a right to choose not only how they live but how they die (P133, social worker).
	Members of MDTs provide support to each other in the context of MAiD cases	I think it would be important to debrief with clinicians on the emotional burden of caring for patients pursuing MAID. I can imagine working with such a heavy caseload may lead to compassion fatigue (P3, social worker). It may also be helpful to prepare by having a discussion with a trusted colleague to seek advice/support (P2, social worker). I would also be mindful to connect with other members of the team on a case like this, to ensure a collaborative care plan could be built around the patient's needs and values (P32, chaplain)
	Standardization within healthcare systems for addressing MAiD and related workflow	I think it can be advantageous (for the pt and clinicians) if we had some sort of checklist or verbiage to guide the clinician through a thorough assessment that helps us to better understand the meaning behind the pt.'s request. Also, utilizing social work to provide resources for organizations that have a vast understanding of the multi‐state process around MAID (P61, social worker). Our hospital has a very clear process for assisting patients with MAiD and the SW team does the paperwork and organization (P7, social worker). At the Veteran's Administration we are limited in our discussions about MAID. However the topic does come up. We talk about the feelings that the veteran may be feeling, the VA's position however share how a veteran can seek out further information (P134, social worker)
4. Person‐centered care that de‐stigmatizes MAiD	Normalize MAiD option/decision	Normalizing and validating their desire to seek out information about this process. Asking open‐ended questions to explore their understanding of MAID; leading with curiosity (P61, social worker). Normalizing the feeling of wanting MAID (P8, chaplain). The most challenging is I talking with another person about taking their own life and normalizing something that is not normal (P11, chaplain)
	Respect and advocate for patient autonomy	Everyone's role is to listen to, advocate for, and help the patient understand and process what it is they need to do to maintain the quality of life they desire (P154, nurse). All have a curious mindset and help patients achieve their goals, values, and preferences (P17, nurse). Advocate for their rights, communication with team, time and space to process, ask questions, reflect, support their family (P33, nurse)
	Support patient and family in context of existential pain	This is a very interdisciplinary topic that should include spiritual and emotional support as well as physical. There may be loose ends, logistical concerns that need to be dealt with and of course existential issues that could be addressed (P95, social worker). I want to understand what the three symptoms “mean” to Reese and explain that when living with a terminal illness “hard lines” can become more fluid with the support of medical professionals who can help mitigate physical symptoms, address loss of control and identity, and explore existential pain (P70, social worker). The most challenging part of MAID conversations has been when a patient describes extreme demoralization and existential pain in these cases it is a matter of supporting them and determining appropriateness of MAID (P20, social worker)

*Note:* References to “MAID” in participant responses are references to “medical aid in dying,” although the acronym is different than “MAiD” used throughout the rest of the paper.

### 3.1 *Theme 1:* Addressing Complexity of MAiD in GBM

Study participants reported that the comprehensive expertise of the MDT should be leveraged to conduct robust, multidisciplinary assessments and offer similar, all‐encompassing support to address the complexity of MAiD in the context of GBM. Participants from all disciplines spoke to the importance of *supporting patients' conceptual understanding of MAiD*. Clinicians highlighted that when patients expressed curiosity about MAiD as a care option, it was critical for them to both assess the patient's current knowledge about MAiD and develop their understanding about MAiD as a care option in service of supporting their ultimate decision. Participants often mentioned “presenting all information about MAiD” and other EOL care options to support informed decision making. Notably, even participants working in settings where MAiD was not a care option for patients described their emphasis on supporting patient understanding, like P101, a nurse: “I work in a hospital setting and we don't participate in MAiD in the hospital, but I always give patients information on how they can pursue MAiD if that's what they desire.”

Clinicians also referenced the need to conduct an *assessment of fears and fear of the future* with their patients considering MAiD. In many responses, clinicians referenced the roles of death anxiety, fear of death, and fear of the dying process in motivating a patient's MAiD decision. As a result, clinicians reported that fully exploring the extent of this fear and its role in patient decision making would be high priority. For example, P134, a social worker, stated: “there can be many fears around what the last chapter of life looks like prior to EOL, and we have also found that if we talk about the fears this may allow for an openness and the meaning of embarking on that part of the journey.” Participants noted the ways in which their exploration of patient fear may lead a patient to move toward or away from MAiD more confidently.

Lastly, participants reported that it was critical for them to *understand drivers of MAiD interest/preference* for patients with cancer. They stated that when patients mentioned the idea of pursuing MAiD or asked for more information about the MAiD process, it was essential for the care team to talk with them in greater detail about why they were considering MAiD as an EOL care option and how they believed it would support their EOL care wishes. Participants often described how they would ask questions to assess specific drivers, like P74, whose professional discipline is unknown, who said they would ask, “What are the specific symptoms or concerns do [sic] you have? If those concerns may be managed would you still like to continue with MAiD? If the symptoms of concern are temporary, would you like to continue? Do you know what MAiD entails?” Clinicians stated that they often asked patients about topics such as wanting “control over one's body and illness” and “fear of not feeling like themselves” as possible reasons for being interested in MAiD. For example, one participant directly stated: “It is meaningful to have these conversations because I can see that a patient is trying to get control in a place that normally they do not have control” (P151, social worker).

### 3.2 *Theme 2:* Thorough Palliative Care Assessment

Participants emphasized *that thorough palliative care assessments* are essential for supporting patients who are thinking about MAiD. They stressed that such assessments help uncover any unmet symptom needs and can make a significant difference in improving quality of life for patients. Clinicians noted that severe symptoms that feel out of control to patients can wear down patients and families and may even make everyday life feel unbearable, leading to some patients considering MAiD earlier than they otherwise would (e.g., P87, chaplain, stated: “The team should be aware of the patient's likelihood of total pain and how the source of the patient's pain is causing them distress”). As a result, exploring the extent of symptom burden and identifying ways to alleviate symptoms were seen as critical by clinicians. Some participants even referenced the value of a palliative care assessment in bringing multidisciplinary teams together, like P116, a nurse: “I would ask more detailed symptom management questions and suggest we form a collaborative team to specifically discuss concerns including patient's Med Onc (medical oncology), Pall Onc (palliative oncology), family and RN (nurse) and to form a plan together that provides for current needs, as well as address patient's future wishes.”

Assessment of patients' *prognostic understanding* was also described as a key component of care (e.g., “I would ask Reese what he/she knows about his/her diagnosis or prognosis,” P121, nurse). Clinicians reported that it was important to evaluate not only how well patients understood their disease trajectory, but also their awareness of existing healthcare options, such as palliative care, hospice, and comfort‐focused treatments. Clarifying prognosis and available supports helped ensure that MAiD requests were grounded in an informed understanding of alternatives.

*Psychosocial assessments* were widely referenced as a means of understanding patients' support networks, emotional needs, and capacity to maintain meaningful daily life. Clinicians described asking open‐ended questions about patients' joys, values, and support systems, and offering guidance around mobilizing assistance or having difficult conversations with loved ones. These efforts were described as key to surfacing sources of distress and identifying actionable opportunities for enhancing quality of life.

The last core tenet of a thorough palliative care assessment as highlighted by clinicians was the value of *exploring spiritual and existential concerns* with patients. Asking about religious beliefs, sources of meaning, and spiritual distress was described as a way to better understand the full scope of a patient's suffering. Participants emphasized the importance of listening with care and curiosity, validating patients' emotional experiences, and creating space for reflection and hope.

### 3.3 *Theme 3:* Strategies for Clinicians and Healthcare Systems to Optimize MAiD Discussions

Many respondents emphasized the importance of *cultivating self‐awareness* when engaging in MAiD‐related discussions, recognizing that their own assumptions could unintentionally shape the tone and content of conversations about MAiD. Many described the value of intentionally grounding themselves prior to conversations, setting aside personal beliefs (e.g., “a very experienced psych RN told me to ‘leave my shit at the door’…” P158, nurse), and approaching patients with openness and emotional neutrality. Clinicians reported that checking in on their own reactions and maintaining a posture of curiosity supported their ability to hold space for the patient and enhanced their work as members of the MDT. One participant even reported that their facility “have [sic] a couple clinicians who opt out because of personal beliefs. If that happens, one of our other physician teams absorbs that patient to see that process through” (P100, nurse). Thus, responses underscored the utmost importance of ensuring that individual value systems do not conflict with patient beliefs and decision‐making processes around MAID via intentional self‐awareness.

*Increased education and training* around MAiD processes also emerged as a key strategy for improving the quality of conversations with patients and families and more effective collaboration with other clinicians on the MDT. Clinicians highlighted gaps in knowledge among clinical staff and expressed a desire for ongoing opportunities to build fluency in MAiD policies, eligibility criteria, and communication practices. Participants emphasized that staff across disciplines should receive role‐specific training to feel more competent in responding when patients raise MAiD as an option and to facilitate more effective collaboration within the MDT.

Clinicians also stressed the emotional and professional importance of *peer support* in the context of MAiD. Many clinicians described the need to debrief difficult cases with colleagues, both to process emotional burdens and to prepare for future discussions. Participants pointed to the importance of leveraging input, insights, and collaboration from their colleagues in order to maximize their impact on patient care and optimize their performance as clinicians.

Finally, participants pointed to the value of *standardization within healthcare systems* to support MAiD‐related workflows. Suggestions included structured assessment tools, clear institutional policies, and defined roles for social work or care coordination teams to handle logistics of MAiD referrals and information distribution. Clinicians noted that when systems have well‐defined protocols for MAiD, both patients and clinicians benefit from greater clarity, continuity, and access to appropriate resources.

### 3.4 *Theme 4:* Person‐Centered Care That De‐Stigmatizes MAiD

Study participants spoke to the importance of *normalizing MAiD* as an option for EOL care and honoring MAiD as a viable choice in the context of patient decision making. Clinicians suggested strategies such as validating patients' desire to explore MAiD as a possibility for future care and described using open‐ended questions and reflective listening to better understand the patient's motivations toward MAiD. Clinicians also highlighted the importance of language that creates space for exploration, referencing the importance of being *curious, non‐judgmental, and respectful of patient values*. Clinicians were confident that advocating for their patients' preferences and respecting their patients' values and care preferences related to MAiD was of utmost importance, and that being on the same page as other members of the MDT about supporting patient autonomy is critical.

Throughout their responses, participants also noted the importance of supporting patient and family needs in the context of *existential pain and suffering*. Clinicians noted not only the importance of delivering person‐centered emotional and spiritual care but also leveraging the range of expertise of the MDT to assess and intervene on existential suffering. Participants reported attunement with the common relationship between patient interest in MAiD and illness‐related existential suffering and described how they would navigate these issues in clinical practice with other colleagues on the MDT.

## 4 Discussion

This qualitative study explored multidisciplinary cancer care clinicians' perspectives on communication priorities and practices in the context of a hypothetical case scenario in which a patient with GBM expressed interest in medical aid in dying (MAiD). Through analysis of responses from 160 participants, four key themes emerged: (1) addressing complexity of MAiD in GBM, (2) thorough palliative care assessment, (3) strategies for clinicians and healthcare systems to optimize MAiD discussions, and (4) the importance of person‐centered care that actively de‐stigmatizes MAiD. Together, these themes highlight the nuanced, layered nature of clinical engagement with MAiD in oncology settings and underscore both practical and ethical considerations that clinicians face, both as individual clinicians and as members of MDTs. Our results highlight that many best practices known to palliative care function similarly in the context of MAiD discussions. However, results from this work also point to the distinct, complex nature of MAiD conversations and the specific ways in which clinicians must navigate them with patients to achieve high‐quality, person‐centered care [27].

In the current study, clinicians emphasized the importance of multidisciplinary assessments as foundational to navigating MAiD discussions with cancer patients and their families. The complexity of GBM—including symptoms of cognitive decline and existential distress—necessitates coordinated input from multiple disciplines. This reflects growing recognition of MAiD decision making in the context of GBM not simply as a singular event but as a complex process that intersects with a patient's evolving physical, psychological, and existential needs [35]. Participants frequently referenced the relevance of input from multiple disciplines—including palliative care, social work, and chaplaincy—to ensure a comprehensive understanding of the patient's condition, prognosis, capacity, and motivation for requesting MAiD. This multidisciplinary lens was seen as essential for both appropriate clinical evaluation and for supporting staff who may face emotional or ethical tension around participation in MAiD. Participant responses highlight not only the core fabric of MAiD assessments for patients with cancer, but also the inherent necessity of multidisciplinary input and oversight for patients considering MAiD as an EOL care option [36].

The importance of comprehensive palliative care assessments as part of MAiD conversations and decision making emerged from participant responses as a distinct yet closely related theme. Clinician responses suggested that a thorough palliative evaluation should be incorporated into any MAiD request, both to ensure that symptom burden is being adequately addressed and to give patients access to, and comprehensive knowledge about, the full range of supportive care options [37]. This finding aligns with previous research suggesting that early integration of palliative care can clarify patient goals and reduce suffering, potentially influencing decisions around MAiD. Further, exploring and addressing uncontrolled symptoms and existential distress may help patients clarify whether MAiD truly aligns with their values and goals. However, participants also recognized that palliative care involvement should not be framed as a barrier or condition to accessing MAiD, but rather as a parallel and complementary process. Participant responses in the current study referencing the importance of patient education about palliative care underscored clinicians' perceived importance of ensuring that patients electing MAiD were provided with access to effective symptom management and care coordination prior to finalizing their decision [38].

Person‐centered care is undoubtedly a tenet of all high‐quality, efficacious palliative care for individuals affected by cancer [39]. However, in the current study, participant responses pointed to the need for exceptionally compassionate, clear, and nonjudgmental communication with patients about MAiD. Clinicians' descriptions of the importance of responding promptly and respectfully to patient inquiries about MAiD highlighted an awareness among clinicians that MAiD continues to be stigmatized, even in jurisdictions where it has been deemed legal and ethical. The fact that participants acknowledged that MDT members might experience moral opposition to MAiD underscored the importance they placed on maintaining a curious attitude free of judgment or personal values. These findings align with broader ethical guidance for best practices and patient‐centered care in medicine, including striking a balance of supporting patient autonomy while maintaining a therapeutic alliance [38, 40].

Though it is well understood that MAiD legislation is still in its infancy in many regions of the United States as well as across the world [7], our results shed light on ethical issues faced by clinicians who lack clear guidance about how to discuss MAiD with their patients, both in participating areas and non‐participating areas. Participants stressed the need for system‐wide standardization of MAiD workflows, both within their institutions and at the state level, noting that variability in institutional policy, documentation procedures, and role clarity can cause confusion across the MDT. Ultimately, participants underscored the reality that effective healthcare system infrastructure could make the difference between enabling or hindering patient‐centered MAiD support. Some participants also emphasized their dependence on outside resources (e.g., non‐profits) to support them with providing patients with information about MAiD: in other words, clinicians were outsourcing beyond their healthcare systems and cancer centers to meet patient needs. As a result, our findings point to the need for prioritizing logistical and workflow changes related to MAiD, both in terms of educating patients about their eligibility and streamlining workflows and decision‐making protocols for oncology care clinicians.

### 4.1 Study Strengths and Limitations

This investigation of multidisciplinary clinicians' perceptions of priorities in MAiD communication with MDTs holds many strengths. First, this work leverages qualitative data representing multidisciplinary clinicians' experiences conducting MAiD discussions across the United States, across jurisdictions with and without legislation legalizing MAiD. Thus, this work contributes novel understandings around clinicians' perceptions of priorities in MAiD conversations with patients as delivered by MDTs with variable access to MAiD in their hospitals and health care systems.

This work is also marked by a few weaknesses. Importantly, our sample, while comprised of multidisciplinary clinicians in oncology, did not include physicians or clinicians in other clinical care settings. As a result, our data lack the input and perspectives of oncologists, palliative care physicians, primary palliative care providers (e.g., primary care physicians, other specialty care providers), and other specialty care clinicians. However, because most MAiD research over the last decade has emphasized the perspectives of physicians [41], including oncologists, our study was unique in its focus on non‐physician clinicians. Further, because patients with cancer are the most common recipients of MAiD, we believe that our results will offer an important contribution to clinical knowledge in terms of how to support this ever‐growing patient population. Additionally, while our study methodology permitted many responses from diverse clinicians, an alternate methodological approach (e.g., semi‐structured interviews) may have resulted in longer, more comprehensive participant responses, which may have produced more robust findings and recommendations for clinical practice. We recommend that future qualitative work in this domain incorporate interviews with clinicians about their perspectives on multidisciplinary care in MAiD. Lastly, we collected data from two different cohorts of the ICC training curriculum: One group of clinicians responded to the survey immediately at the end of the course, while the other group responded at 12 months post course. Although it is possible that participants who responded nearly 1 year after completing the course had more time to reflect on their experiences in practice and may have responded differently, each course has very minor content related specifically to communication surrounding MAiD. Therefore, it is unlikely that participant responses by cohort varied or were discrepant based on time elapsed since participation in the ICC course.

### 4.2 Clinical Implications

Our findings suggest several actionable insights for improving care delivery in MAiD conversations with cancer patients. The emphasis of participant responses on the importance of MDT subspecialties in addressing the complexity of MAiD conversations in GBM highlights that no single discipline can adequately address the complexity of MAiD decisions and underscores the ongoing cultural shift in palliative care to elevate the role of social workers, chaplains, and other non‐medical professionals in EOL communication. As a result, findings underscore the importance of offering specialized training and support for non‐physician specialists surrounding MAiD in order to maximize opportunities for individuals to receive whole‐person, evidence‐based care. Our findings also underscore a clear and ongoing need to broaden public awareness about the full spectrum of EOL care options, including palliative care, hospice, and comfort‐focused approaches—not only to ensure that patients make informed decisions, but also to counter the misconception that suffering is inevitable or that MAiD is the only pathway to relief. Participant responses indicated revealed their heightened attentiveness to evaluating patient existential suffering, as well as their spiritual and existential beliefs. Embedded within these discussions is an inherent attentiveness to deficits in patient well‐being that often drive interest in MAiD but that could be ameliorated with the appropriate supports in place. This endeavor includes ensuring that symptom management is proactively addressed and optimized for all patients, regardless of their care preferences.

## 5 Conclusions

This study offers insight into how multidisciplinary oncology teams navigate the evolving landscape of MAiD conversations for patients with aggressive cancers. While many best practices from palliative care remain applicable, clinicians described MAiD discussions as uniquely complex, requiring intentional communication, system‐level clarity, and a commitment to patient‐centered care. The findings underscore the importance of comprehensive, interdisciplinary assessments; early integration of palliative care; and the cultivation of nonjudgmental, well‐informed dialog with patients. Above all, findings emphasize that person‐centered, non‐stigmatizing multidisciplinary communication is foundational to patient support. As guidelines and legislation for MAiD evolve, healthcare systems should prioritize provider education and institutional workflows that empower clinicians to support patients through all EOL care options.

## Author Contributions

**Meghan McDarby:** conceptualization (equal), formal analysis (lead), methodology (equal), writing – original draft (lead), writing – review and editing (lead). **Alix Youngblood:** formal analysis (equal), writing – original draft (lead), writing – review and editing (lead). **Megan Miller:** conceptualization (equal), writing – review and editing (equal). **William E. Rosa:** conceptualization (equal), methodology (equal), supervision (equal), writing – original draft (equal), writing – review and editing (equal). **Haley Buller:** investigation (lead), methodology (lead), project administration (lead), supervision (equal), writing – review and editing (equal). **Betty R. Ferrell:** conceptualization (lead), funding acquisition (lead), investigation (lead), methodology (lead), resources (lead), supervision (lead), writing – review and editing (equal).

## Funding

This work was supported by the National Institute of Health/National Cancer Institute (Ferrell, PI, R25CA240111, Interdisciplinary Communication Training in Oncology. 03/6/2020–03/5/2025 NIH/NCI). Meghan McDarby is supported by T32 CA00946 and P30 CA008748. Megan Miller received research support from the University of Wisconsin‐Madison, Office of the Vice Chancellor for Research and Graduate Education with funding from the Wisconsin Alumni Research Foundation.

## Ethics Statement

Study recruitment and procedures were deemed exempt by the City of Hope IRB because participant data could not be linked to individual respondents (assurance number 00000692).

## Conflicts of Interest

The authors declare no conflicts of interest.

## Data Availability

The data that support the findings of this study are available from the corresponding author upon reasonable request.

## References

[1] “Choreographing Death: A Social Phenomenology of Medical Aid‐in‐Dying in The United States – Buchbinder,” (2018), https://anthrosource.onlinelibrary.wiley.com/doi/abs/10.1111/maq.12468. 10.1111/maq.12468 30014621

[2] “Supplement Section I: The Survey IAHPC,” (2023), https://iahpc.org/resources/publications/euthanasia‐and‐physician‐assisted‐suicide/special‐issue‐on‐assisted‐dying‐practices/methodology‐and‐key‐findings/.

[3] J. Downar , S. Green , D. Hoffman , and T. M. Pope , “Sorry, Not Sorry: Canadian MAID Is Voluntary, Safe, Carefully Regulated, and Valued,” Am J Bioeth AJOB 25, no. 5 (2025): 33–35, 10.1080/15265161.2025.2488291. 40339094

[4] J. Downar , S. MacDonald , and S. Buchman , “Medical Assistance in Dying and Palliative Care: Shared Trajectories,” Journal of Palliative Medicine 26, no. 7 (2023): 896–899, 10.1089/jpm.2023.0209. 37428971

[5] R. Gallagher , R. Coelho , P. D. Violette , K. S. Gaind , and H. M. Chochinov , “Response to Medical Assistance in Dying, Palliative Care, Safety, and Structural Vulnerability,” Journal of Palliative Medicine 26, no. 12 (2023): 1610–1617, 10.1089/jpm.2023.0581. 37955548 PMC10714107

[6] “Death with Dignity U.S. Legislative Status State Map,” (2025).

[7] E. Kozlov , M. Nowels , M. Gusmano , M. Habib , and P. Duberstein , “Aggregating 23 Years of Data on Medical Aid in Dying in the United States,” Journal of the American Geriatrics Society 70, no. 10 (2022): 3040–3044, 10.1111/jgs.17925. 35708060 PMC9588508

[8] T. M. Pope , “Medical Aid in Dying: Key Variations Among U.S. State Laws,” Social Science Research Network 14 (2020): 3743855, 10.2139/ssrn.3743855.

[9] L. Watson , C. Link , S. Qi , et al., “Symptom Burden and Complexity in the Last 12 Months of Life Among Cancer Patients Choosing Medical Assistance in Dying (MAID) in Alberta, Canada,” Current Oncology 29, no. 3 (2022): 1605–1618, 10.3390/curroncol29030135. 35323335 PMC8947648

[10] G. Chandhoke , G. Pond , O. Levine , and S. Oczkowski , “Oncologists and Medical Assistance in Dying: Where Do We Stand? Results of a National Survey of Canadian Oncologists,” Current Oncology 27, no. 5 (2020): 263–269, 10.3747/co.27.6295. 33173378 PMC7606035

[11] C. Chiang , S. A. Climans , K. Edelstein , and J. A. H. Bell , “Decision‐Making Around End‐Of‐Life Care in Brain Cancer Patients: A Scoping Review,” Ethics Med Public Health 23 (2022): 100778, 10.1016/j.jemep.2022.100778.

[12] A. Nielsen , A. Martinez , and J. Dains , “Cancer Patient Perspectives on Medical Aid in Dying: An Integrative Review,” Cancer Care Res Online 5, no. 2 (2025): e070, 10.1097/CR9.0000000000000070.

[13] B. B. Reeve , A. L. Potosky , A. W. Smith , P. K. Han , R. D. Hays , and W. W. Davis , “Impact of Cancer on Health‐Related Quality of Life of Older Americans,” Journal of the National Cancer Institute 101, no. 12 (2009): 860–868. 19509357 10.1093/jnci/djp123 PMC2720781

[14] D. Osoba , “Measuring the Effect of Cancer on Quality of Life,” in Effect of Cancer on Quality of Life (CRC Press, 1991).

[15] C. Thabet , P. Wheatley‐Price , and S. Moore , “Medical Assistance in Dying in Patients With Cancer,” JCO Oncol Pract 19, no. 9 (2023): 819–827, 10.1200/OP.22.00615. 37582243

[16] M. Li , G. K. Shapiro , R. Klein , et al., “Medical Assistance in Dying in Patients With Advanced Cancer and Their Caregivers: A Mixed Methods Longitudinal Study Protocol,” BMC Palliative Care 20, no. 1 (2021): 117, 10.1186/s12904-021-00793-4. 34289838 PMC8296526

[17] A. L. Back , E. K. Fromme , and D. E. Meier , “Training Clinicians With Communication Skills Needed to Match Medical Treatments to Patient Values,” Journal of the American Geriatrics Society 67, no. S2 (2019): S435–S441, 10.1111/jgs.15709. 31074864

[18] J. Brown , D. Goodridge , A. Harrison , J. Kemp , L. Thorpe , and R. Weiler , “Care Considerations in a Patient and Family‐Centered Medical Assistance in Dying Program,” Journal of Palliative Care 37, no. 3 (2022): 341–351, 10.1177/0825859720951661. 32854581

[19] B. M. Hales , S. Bean , E. Isenberg‐Grzeda , B. Ford , and D. Selby , “Improving the Medical Assistance in Dying (MAID) Process: A Qualitative Study of Family Caregiver Perspectives,” Palliative & Supportive Care 17, no. 5 (2019): 590–595. 30887936 10.1017/S147895151900004X

[20] A. Ho , J. S. Norman , S. Joolaee , K. Serota , L. Twells , and L. William , “How Does Medical Assistance in Dying Affect End‐Of‐Life Care Planning Discussions?,” Experiences of Canadian Multidisciplinary Palliative Care Providers 15 (2021): 26323524211045996. 10.1177/26323524211045996 PMC8458666 34568826

[21] Y. J. Liu , L. P. Wu , H. Wang , Q. Han , S. N. Wang , and J. Zhang , “The Clinical Effect Evaluation of Multidisciplinary Collaborative Team Combined With Palliative Care Model in Patients With Terminal Cancer: A Randomised Controlled Study,” BMC Palliative Care 22, no. 1 (2023): 71, 10.1186/s12904-023-01192-7. 37312118 PMC10262549

[22] M. Taberna , F. Gil Moncayo , E. Jané‐Salas , et al., “The Multidisciplinary Team (MDT) Approach and Quality of Care,” Frontiers in Oncology 10 (2020): 85, 10.3389/fonc.2020.00085. 32266126 PMC7100151

[23] B. S. Leclerc , L. Blanchard , M. Cantinotti , et al., “The Effectiveness of Interdisciplinary Teams in End‐Of‐Life Palliative Care: A Systematic Review of Comparative Studies,” Journal of Palliative Care 30, no. 1 (2014): 44–54, 10.1177/082585971403000107. 24826443

[24] T. Soukup , B. W. Lamb , A. Morbi , et al., “Cancer Multidisciplinary Team Meetings: Impact of Logistical Challenges on Communication and Decision‐Making,” BJS Open 6, no. 4 (2022): zrac093, 10.1093/bjsopen/zrac093. 36029030 PMC9418925

[25] T. R. Rasmussen , A. Gouliaev , E. Jakobsen , et al., “Impact of Multidisciplinary Team Discrepancies on Comparative Lung Cancer Outcome Analyses and Treatment Equality,” BMC Cancer 24, no. 1 (2024): 1423, 10.1186/s12885-024-13188-4. 39558297 PMC11575113

[26] A. Ho , S. Joolaee , K. Jameson , and C. Ng , “The Seismic Shift in End‐of‐Life Care: Palliative Care Challenges in the Era of Medical Assistance in Dying,” Journal of Palliative Medicine 24, no. 2 (2021): 189–194. 32584638 10.1089/jpm.2020.0185

[27] E. Wiebe , S. Green , and K. Wiebe , “Medical Assistance in Dying (MAiD) in Canada: Practical Aspects for Healthcare Teams,” Ann Palliat Med 10, no. 3 (2021): 3, 10.21037/apm-19-631. 32787380

[28] D. Sipos , B. L. Raposa , O. Freihat , et al., “Glioblastoma: Clinical Presentation, Multidisciplinary Management, and Long‐Term Outcomes,” Cancers 17, no. 1 (2025): 17010146, 10.3390/cancers17010146. PMC11719842 39796773

[29] C. McKinnon , M. Nandhabalan , S. A. Murray , and P. Plaha , “Glioblastoma: Clinical Presentation, Diagnosis, and Management,” BMJ 374 (2021): n1560, 10.1136/bmj.n1560. 34261630

[30] F. W. Boele , S. Butler , E. Nicklin , et al., “Communication in the Context of Glioblastoma Treatment: A Qualitative Study of What Matters Most to Patients, Caregivers and Health Care Professionals,” Palliative Medicine 37, no. 6 (2023): 834–843, 10.1177/02692163231152525. 36734532 PMC10227096

[31] B. Ferrell , “National Consensus Project Clinical Practice Guidelines for Quality Palliative Care: Implications for Oncology Nursing,” Asia‐Pacific Journal of Oncology Nursing 6, no. 2 (2019): 151–153, 10.4103/apjon.apjon_75_18. 30931359 PMC6371670

[32] W. E. Rosa , M. McDarby , H. Buller , and B. R. Ferrell , “Communicating With LGBTQ+ Persons at End of Life: A Case‐Based Analysis of Interdisciplinary Palliative Clinician Perspectives,” Psycho‐Oncology 32, no. 12 (2023): 1895–1904, 10.1002/pon.6237. 37929880 PMC10842027

[33] B. R. Ferrell , M. L. Twaddle , A. Melnick , and D. E. Meier , “National Consensus Project Clinical Practice Guidelines for Quality Palliative Care Guidelines,” Journal of Palliative Medicine 21, no. 12 (2018): 1684–1689, 10.1089/jpm.2018.0431. 30179523

[34] W. F. Owen , “Interpretive Themes in Relational Communication,” Quarterly Journal of Speech 70, no. 3 (1984): 274–287, 10.1080/00335638409383697.

[35] M. Simpson‐Tirone , S. Jansen , and M. Swinton , “Medical Assistance in Dying (MAiD) Care Coordination: Navigating Ethics and Access in the Emergence of a New Health Profession,” HEC Forum 34, no. 4 (2022): 457–481. 35870101 10.1007/s10730-022-09489-5 PMC9308109

[36] J. J. Sanders , S. Temin , A. Ghoshal , et al., “Palliative Care for Patients With Cancer: ASCO Guideline Update,” Journal of Clinical Oncology 42, no. 19 (2024): 2336–2357, 10.1200/JCO.24.00542. 38748941

[37] E. R. Brassfield and M. Buchbinder , “Clinicians Perspectives on the Duty to Inform Patients About Medical Aid‐In‐Dying,” AJOB Empir Bioeth 11, no. 1 (2020): 53–62, 10.1080/23294515.2019.1695016. 31829903

[38] K. Boström , T. Dojan , C. Rosendahl , L. Gehrke , R. Voltz , and K. Kremeike , “How Do Trained Palliative Care Providers Experience Open Desire to Die‐Conversations? An Explorative Thematic Analysis,” Palliative & Supportive Care 22, no. 4 (2024): 681–689, 10.1017/S1478951522001006. 35942616

[39] A. L. Back , “Patient‐Clinician Communication Issues in Palliative Care for Patients With Advanced Cancer,” Journal of Clinical Oncology 38, no. 9 (2020): 866–876, 10.1200/JCO.19.00128. 32023153

[40] B. Pesut , S. Thorne , M. Greig , A. Fulton , R. Janke , and M. Vis‐Dunbar , “Ethical, Policy, and Practice Implications of Nurses Experiences With Assisted Death: A Synthesis,” ANS. Advances in Nursing Science 42, no. 3 (2019): 216–230, 10.1097/ANS.0000000000000276. 31335329 PMC6686960

[41] J. K. Fujioka , R. M. Mirza , P. L. McDonald , and C. A. Klinger , “Implementation of Medical Assistance in Dying: A Scoping Review of Health Care Providers’ Perspectives,” Journal of Pain and Symptom Management 55, no. 6 (2018): 1564–1576.e9, 10.1016/j.jpainsymman.2018.02.011. 29477968

